# Data Resource Profile: Children Looked After Return (CLA)

**DOI:** 10.1093/ije/dyw117

**Published:** 2016-07-13

**Authors:** Louise Mc Grath-Lone, Katie Harron, Lorraine Dearden, Bilal Nasim, Ruth Gilbert

**Affiliations:** ^1^ Administrative Data Research Centre for England,; ^2^ University College London Institute of Child Health,; ^3^ London School of Hygiene & Tropical Medicine,; ^4^ UCL Institute of Education and; ^5^ Institute for Fiscal Studies, London, UK

**Keywords:** Looked-after children, children in care, childhood adversity, administrative data

## Data resource basics

### Childhood adversity

Early exposure to adversity, such as abuse or neglect, is associated with poorer outcomes across social, education and health domains. ^1^^,^^2^ Children in care (referred to as looked-after children in the UK ^3^ ) are a vulnerable group who experience adversity serious enough for the state to intervene in family life and place them under the supervision of child protection services within the home or, more frequently, to remove the child and place them in out-of-home care (OHC). ^4^ In England, placement in OHC can be voluntary (i.e. with parental consent) or mandated by a court. Some looked-after children have complex health needs and are voluntarily placed in temporary care in order to provide respite to their parents, ^5^ but the majority of children in OHC are removed from their parents for reasons related to abuse or neglect. ^6^

Being in OHC is an indicator of serious childhood adversity and a predictor of future adverse health, education and social outcomes. ^7^ For example, children in OHC have poorer mental and physical health than their peers, ^8–10^ are more likely to engage in risky behaviours such as smoking, drinking and drug-taking ^11^ and have higher rates of teenage pregnancy and premature death. ^12^ The causes of these adverse outcomes are complex and there is considerable heterogeneity among looked-after children. ^13–15^ Some variation in outcomes has been associated with key characteristics of the care children receive while being looked after (e.g. age at first entry, setting, duration, stability) or with their exit from the social care system (e.g. destination, re-entry). ^16–18^ For example, children in foster care have better mental health outcomes than those in residential group care, ^11^ and psychiatric disorders are more common among children who experience multiple placement moves. ^10^ It is therefore important to determine the prevalence among the child population of being placed in OHC and to explore how different types or patterns of care are associated with outcomes, both in childhood and in later life.

Many studies of looked-after children in the UK are based on surveys; ^8–10^ however, these may have selection and/or recall biases, and an alternative administrative data source that can be used is the Children Looked After Return (CLA). The CLA offers an important resource to improve understanding about the characteristics of children placed in OHC, how patterns of care vary across the country and are changing over time, and the relationships between the type or pattern of care and subsequent outcomes.

### Purpose and scope of the Children Looked After Return

In England, children’s social care services are delivered at local government level (i.e. by local authorities). The CLA is a national individual-level dataset held by the Department for Education (DfE), which contains information on all looked-after children and recent care leavers in England. Data collection began in 1992 ( Table 1 ) and is ongoing via an annual online census of local authorities. Initially, data collection was mandated for all children in England who were looked after in the year ending 31 March 1992; however, between 1998 and 2003 it was restricted to a one-third sample (selected as children with a day of birth divisible by three) before reverting to include all looked-after children in 2004. The CLA contains detailed care histories for looked-after children including the start and end dates of each episode of OHC.

**Table 1.** Coverage of data in the Children Looked After Return (1992 to 2016)

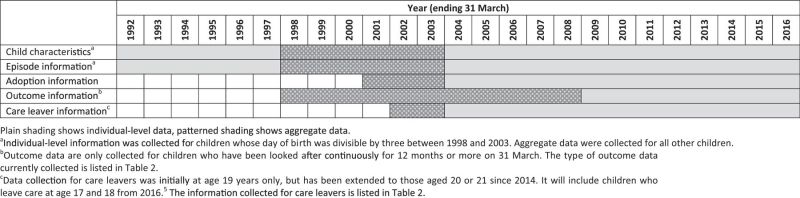

According to the DfE, the purpose of the CLA is to monitor the care and outcomes of looked-after children (while in care and on reaching adulthood) and to enable evaluation of the potential effects of government policy initiatives. ^5^ Outcome data were first collected in 1999, but were limited to the activity of children in care at age 16 years (i.e. taking exams, in further education, working, etc.). Since 2009, the outcome data collected by local authorities have been expanded to include child-level information on health-related outcomes such as immunizations, health checks and Strengths & Difficulties Questionnaire (SDQ) scores. However, outcome data are only collected for children in continuous care for 12 months or more. In 2002, collection of data on the activity and accommodation of care leavers at age 19 began with further follow-up at other ages introduced in later years.

All looked-after children are included in CLA (with the exception of the previously-described sample restrictions between 1998 and 2003). However, the CLA does not include private fostering arrangements in which a child is cared for by an adult who is not a close relative (i.e. someone other than a parent, grandparent, sibling, aunt or uncle). ^19^ The most recent CLA for the year ending 31 March 2015 contained details of 99 230 looked-after children—the highest figure since 1985. ^20^ Coverage of care leavers in CLA is not complete; information was collected for 84% ( *n* = 22 510) in 2014 and 88% ( *n* = 23 170) in 2015. ^20^

## Data collected

### Dataset production

Each year, all 150 local authorities in England must submit details of the looked-after children in their area and the care provided to them during the period 1 April to 31 March to the DfE, via an online census. The number of children in care varies from year to year, but in the most recent period of data collection (1 April 2014 to 31 March 2015) data were collected for 99 230 children. Local authorities must also provide information for specific groups of care leavers (i.e. young people who were looked after as adolescents and whose 19th to 21st birthday occurred during the preceding year). Data must be returned and checked by local authorities before the end of June. ^5^ A national dataset is then collated by DfE. Aggregate tables and summary statistics (at national and local authority levels) are then produced by DfE and published online. ^21^

### Dataset structure: episodes and periods of care

In the CLA, a child’s care record is divided into episodes. An episode is the length of time a child is looked after under the same legal status and in the same placement. When a child’s legal status and/or placement changes, a new episode begins. ^5^ The start and end date of each individual episode is recorded in the CLA, and an episode cannot be less than 24 h. Episodes of care can be in the home (under supervision) or in alternative out-of-home accommodation (e.g. with a foster carer or in a children’s home, Supplementary Table 1 , available as Supplementary data at *IJE* online) and can be voluntary or legally mandated ( Supplementary Table 2 , available as Supplementary data at *IJE* online). A period of care is the time that a child is continuously looked after by a local authority. A period can consist of one or more episodes.

### Measures collected in CLA

The measures collected in CLA have changed over time but can be broadly grouped as child characteristics, episode information, and indicators and outcomes of care ( Table 2 ).

**Table 2.** Measures collected by Children Looked After Return

Child characteristics	Episode information	Indicators and outcomes of care	
For all children in care Child ID Gender Date of birth Ethnicity Unique Pupil Number Is a girl in care a mother? Is the child in care an unaccompanied asylum seeker?	For all children in care Local authority providing care Start date of care episodes Reason a new episode started Reason a placement changed Legal status of child Category of need of child Placement type Placement location ( in/outside local authority ) Placement provider ( local authority, voluntary sector, etc. )* Unique Reference Number of placement provider End date of care episodes Reason episode ceased For children in continuous care for 12 months Home postcode when entering care Placement postcode Distance between placement and home	For all children in care Start and end dates of any period that child was missing from care Is the child re-entering care after the breakdown of a permanent placement? For children re-entering care after the breakdown of a permanent placement Type of permanent arrangement Date of exit to this permanent arrangement The local authority where the child was previously looked after For children in continuous care for 20 days Date of statutory review Child’s participation in the review For children who are placed for adoption Date the decision to be placed for adoption was made Date child and prospective adopters were matched Date of adoption Number of prospective adopters Gender of prospective adopters Marital status of prospective adopters Date decision that a child should no longer be placed for adoption was made Reason why a child should no longer be placed for adoption	For children in continuous care for 12 months Was the child convicted during the year (if aged > 10 years)? Are health surveillance checks up to date (if aged < 5 years)? Are immunizations up to date? Were the child’s teeth checked by a dentist during the year? Are annual health assessments up to date? Was the child identified as having a substance misuse problem? Was the child offered an intervention for substance misuse problem? Was the child eligible to take GCSE examinations? What was the child doing when aged 16 or over (e.g. in school, employment)? Strengths & Difficulties Questionnaire score For care leavers Was the local authority in touch with the young person during the year? What was the child doing on their birthday (i.e. in education, employment)? What type of accommodation was the child living in on their birthday? Was the accommodation suitable?

The years in which these variables were collected in the CLA vary and are described in full in official Department for Education guidance. The underlined variables are available for request from the Department for Education through the National Pupil Dataset team. Other variables are not routinely available to researchers, but can be requested. *Care episodes recorded in CLA are funded by the state via local authorities, but may be delivered on their behalf through approved private organizations (e.g. a looked-after child may be placed with an agency foster carer or in a children’s home run by a charity).

#### Child characteristics

When a child becomes looked after by a local authority for the first time, they are assigned a child ID—the main identifier in the CLA. This allows a child’s record of care to be linked over time and enables longitudinal analyses. The demographic information collected in the CLA is limited to date of birth, gender and ethnicity (18 categories). Names are not collected. Whether a child is an unaccompanied asylum seeker (or a mother, for girls who are looked after) is also recorded, but this information is not routinely available to researchers. A pseudonymized unique pupil number (UPN) is recorded for looked-after children who attend a maintained (or state-funded) school or nursery in England, ^22^ which allows linkage of CLA data to other education and social care datasets held by DfE.

#### Episode information

Detailed information related to each episode of care is collected in the CLA; for example, start and end dates, placement type, location and provider. Placement type describes the setting in which a child is cared for. Children may be placed at home with their parents while being looked after, but the majority are removed and placed in OHC. ^6^ OHC placements include foster care by relatives, friends, strangers or potential adopters; group care in children’s homes, residential schools, care homes or residential units; independent living in a bed and breakfast (B&B), flat or bedsit and ‘other’ settings such as young offender institutes and prisons. The codes used to record placement type have changed over time and are described in Supplementary Table 1 . When a child’s placement changes (even to another placement of the same type) a new episode begins. However, only placements lasting 24 h or more are recorded; therefore if multiple placement changes occur in 1 day, only the final placement is recorded. ^5^

The reason a child becomes looked after is recorded in the CLA as their ‘category of need’. These hierarchical categories are: abuse or neglect, child’s disability, parental illness or disability, family in acute stress, family dysfunction, socially unacceptable behaviour, low income and absent parenting. Though it is likely that a child will become looked after for a combination of the above reasons, only one (the highest ordered in the list) is recorded. ^23^ Before the 1 April 2000, a more detailed variable was used to capture the reason a child was looked after, and the relationship between these former ‘reasons looked after’ and current ‘categories of need’ is described in Supplementary Table 2 .

The legal status of a child describes the legal framework under which a child enters the social care system. For example, child protection is used to ensure the safety of a child who is considered to be in need, and this legal status category includes emergency protection orders and police protection powers (used in urgent cases where rapid intervention is required) and child assessment orders (used in non-emergency cases where there are suspicions but no convincing evidence of actual or likely harm). ^24^ Children can also be looked after voluntarily (i.e. with parental consent) under Section 20 of the Children Act 1989. ^3^ Though it is possible for a child to have multiple legal statuses (e.g. to be under a care order and awaiting trial), only the most recent legal status is recorded in the CLA. As for placement setting, if there are multiple changes in 1 day, only the final legal status is recorded. ^5^ The codes used to record legal status have changed over time and are described in Supplementary Table 3 (available as Supplementary data at *IJE* online).

When a child ceases to be looked after, the reason the period of care ended is recorded. For example, a child may cease to be looked after because they return home to their parents or are adopted. They may also leave care through the granting of a residence or special guardianship order which confers differing levels of parental responsibility to a guardian ^25^ (such as a relative or former foster carer). The codes used to record the reason OHC ceases and how a child exits the social care system are described in Supplementary Table 4 (available as Supplementary data at *IJE* online).

#### Indicators and outcomes of care

One of purposes of the CLA is to monitor outcomes of looked-after children while in care and on reaching adulthood; however, outcomes are generally recorded only for children who have been in continuous care for 12 months or more. The data recorded for these children in long-term care include whether they were convicted of a crime, identified as having a substance misuse problem, offered intervention to treat substance misuse, and had up-to-date health checks, dental examinations and immunizations. Children aged 4 to 16 years should also have an annual SDQ score recorded (which can be used as an indicator of emotional or behavioural disorders). Similarly, outcome data are only collected for ‘relevant and eligible’ care leavers as defined by current DfE guidance, ^5^ i.e. a young person who was looked after at the age of 16 or 17 and had been looked after for at least 13 weeks after the age of 14. The outcomes recorded for care leavers are participation in education and/or employment and living arrangements, currently at age 19 to 21. Indicators of care, such as time to adoption, participation in statutory case reviews and being missing from care, are also collected in CLA. Data related to indicators and outcomes of care are not routinely available to researchers but can be requested.

### Data quality checks

The CLA undergoes a number of automated validation checks when data are being returning by local authorities; ^26^ for example, fields that are blank or contain an invalid value will be flagged for review and correction. Unlikely/impossible sequences of dates or combinations of legal status and placement are also automatically flagged, as is information that contradicts records entered in previous years for the same child. During the validation checks, local authorities may correct errors or update previous years’ data (i.e. enter an end date for an episode of care that had been ongoing at the time of the latest census).

## Data resource use

### Describing trends

DfE statistical tables and reports are published annually and are readily available to the general public online [ https://www.gov.uk/government/organisations/department-for-education/about/statistics ]. These DfE tables include information on the rate of looked-after children in England by local authority, which can be used as an area-level indicator of childhood adversity. CLA data are used to monitor the use of OHC in England and outcomes of looked-after children over time. For example, recent DfE reports indicate that the rates of substance misuse and offending are falling among children in care, ^20^ but the proportion of care leavers not in education, employment or training (NEET) has increased. ^27^

### Monitoring outcomes using linkage

Only limited educational outcomes are recorded in CLA; however, another DfE administrative dataset [(The National Pupil Dataset (NPD)] contains detailed information on a broad range of educational outcomes, including absences, exclusions, Special Educational Needs (SEN) support and type of school attended. Since 2006, NPD and CLA data have been routinely linked via UPN ^28^ by the DfE; this linked dataset has been used to describe the relative educational outcomes for children looked after in continuous care for 12 months or more. ^27^ Pseudonymized linked CLA-NPD data have also been used by researchers. For example, a recent study by Sebba *et al.*^29^ explored the effects of the type and timing of OHC on children’s educational outcomes, specifically the attainment of children eligible to sit GCSE exams in 2013. This study also involved linkage to a third DfE dataset that contains details of children who are referred to social services but are not placed in care (the Children In Need (CIN) dataset), available from 2009. This additional linkage allowed researchers to conduct more granular analyses in terms of exposure to adversity during childhood. Children in care (due to serious adversity) were compared with children in need (who experienced adversity at a level that was insufficient to warrant state involvement) and with all other children who were not in care or in need. The results of these analyses suggest that some of the gap in educational attainment between children in care and their peers can be attributed to differences in deprivation and SDQ scores, but early placement in long-term foster care can have a protective effect on attainment. ^29^ However, this analysis was limited to children in care at the time of sitting their GCSE exams (at age 15/16), and early exposure to care was simplistically defined as being in continuous care for at least 12 months and having also been in care during late primary school years (Key Stage 2).

### Analyses across the child life course

DfE reports make limited use of the rich longitudinal records of care available in CLA. For example, DfE annual reports focus on the number of placement moves a child has during a year, rather than the total number of moves they experience during their total time in care. However, the CLA can also be used to generate evidence on the child life course of care. For example, in a recent study that used longitudinal CLA data, we calculated the proportion of children in England who ever entered care throughout childhood, using synthetic birth cohorts. We found that one in 30 children born 1992–94 (3.3%) had entered OHC by age 18 years, ^30^ a much higher figure than the 0.6–0.9% of children who spend time in care in any given year as reported by the DfE. ^20^ The cumulative proportion of children ever entering care also appeared to be increasing (particularly among infants) and was disproportionately higher among Black, Mixed or Other ethnicity children. Decomposition of these changes over time vis-à-vis concurrent changes in the ethnic composition of the child population indicated that the overall increase in the proportion of children entering care was primarily due to an increase in the proportion of White children entering care, rather than increased ethnic diversity among children in England.

### Cross-national comparisons

Aggregate or child-level CLA data can also be used to explore variation in child protection and social care systems between different countries. For example, Gilbert *et al.* compared trends in the use of OHC among infants in England with five other countries using annual CLA figures published by DfE, ^31^ and Ubbesen *et al.* used individual-level longitudinal CLA data to compare the patterns of entry to care and type of OHC used in Denmark and England. ^32^

## Strengths and weaknesses

### Strengths

The main strengths of the CLA are that it has national coverage and is an administrative dataset, thereby negating issues of recall or selection bias associated with survey-based studies of OHC that rely on self-report by care leavers or caregivers. The CLA has collected cross-sectional data annually since 1992, and this allows for changes over time in the population of looked-after children, and the characteristics of the care they receive, to be reliably described. The CLA is also a longitudinal dataset that contains complete histories for children and allows care trajectories to be explored in detail. An additional strength is that summary statistics are freely available to download online for use as an indicator of local authority-level adversity during childhood. Furthermore, the DfE recognizes the unique value of the CLA as a longitudinal data source for policy evaluation and research and they are committed to continued data collection and improvement of content. For example, permanence is a central component of current social care policy in England; ^33^ therefore, indicators of a breakdown in a permanent exit from care (i.e. adoption, special guardianship order or residence order) and of permanence within social care system via long-term foster care, were recently introduced. ^23^ CLA data can also be used to evaluate local policies as data are available broken down by local authority.

### Weaknesses

The restriction of data collection between 1998 and 2003 limits the power of the longitudinal dataset, particularly when exploring variation by local authority or for relatively rare placements or outcomes (e.g. death). A further limitation is that child ID is a local authority-specific identifier. If a child is looked after in more than one local authority, they will be assigned multiple child IDs, consequently preventing linkage of care records across these administrative boundaries. Similarly, when a child is adopted they receive a new legal identity. Therefore, if they subsequently become looked after again, they are assigned a new child ID. This means that a child’s records of care pre- and post-adoption are not linked. However, the main limitation of the CLA is that (as it is an administrative dataset) it does not contain baseline characteristics of children entering care or their families or provide detailed information related to the care and support looked-after children receive (e.g., interventions provided, parental contact, placement with siblings, etc.). Furthermore, outcome data are only collected for specific groups of looked-after children and care leavers, and linkage to other non-DfE datasets (related to health or justice, for example) is not facilitated as names are not collected.

## Data resource access

Aggregate statistical tables, annual reports and documentation related to CLA are available to the public at [ https://www.gov.uk/search?q = children+looked+after+]. Requests for pseudonymized child-level CLA can be made by researchers through the NPD team at DfE. Data related to child characteristics and episodes of care (underlined in Figure 2) are routinely available for request from 2006 onwards. Other years of data or variables (such as SDQ score, postcode or UPN) are not routinely available, but can be requested and have been supplied to researchers in the past. ^29^ Though CLA data is pseudonymized, it is considered ‘tier 1’ (i.e. sensitive personal information); therefore to obtain an extract, researchers must complete an information security questionnaire and application form, which are considered by an advisory panel. When making an application for CLA data, the need for each requested variable must be clearly justified by researchers. Applications can also be made to link CLA data to NPD and/or CIN data. Further application details and documents are available at [ https://www.gov.uk/guidance/national-pupil-database-apply-for-a-data-extract ].

## Funding

This research was funded by the Economic and Social Research Council, grant reference number ES/L007517/1, establishing the Administrative Data Research Centre for England (ADRC-E). The ADRC-E is led by the University of Southampton and run in collaboration with University College London, the London School of Hygiene & Tropical Medicine, the Institute for Fiscal Studies and the Office for National Statistics (ONS).

**Conflict of interest:** None declared.

**CLA in a nutshell**

- Looked-after children represent a vulnerable population who have encountered serious early adversity and have poorer health, social and educational outcomes than their peers, both in childhood and in later life.
- The Children Looked After Return (CLA) is an administrative dataset routinely collected by the Department for Education in England to monitor outcomes of looked-after children (while in care and on reaching adulthood) and to enable evaluation of the potential effects of government policy initiatives.
- It contains child-level data about all looked-after children in England and recent care leavers, including child characteristics, episodes of care and outcomes.
- Data collection began in 1992, and since then the CLA has been used by researchers to describe the prevalence of children in care and explore their relative educational outcomes through linkage with other datasets.
- Aggregate statistics are publicly available and researchers can apply for pseudonymized, child-level extracts (including linkage to other Department for Education datasets) at [ https://www.gov.uk/guidance/national-pupil-database-apply-for-a-data-extract ].

## Supplementary Material

Supplementary Data

## References

[1] Anda RF Felitti VJ Bremner JD . The enduring effects of abuse and related adverse experiences in childhood . Eur Arch Psychiatry Clin Neurosci 2006 ; 256**:** 174 – 86 . 16311898 10.1007/s00406-005-0624-4 PMC3232061

[2] Shonkoff JP Garner AS Siegel BS . The lifelong effects of early childhood adversity and toxic stress . Pediatrics 2012 ; 129**:** e232 – 46 . 22201156 10.1542/peds.2011-2663

[3] UK Government . Children Act 1989 (UK) . London : Stationery Office , 1989 .

[4] Daniel B . Concepts of adversity, risk, vulnerability and resilience: a discussion in the context of the ‘child protection system.’ Soc Policy Soc 2010 ; 9**:** 0 7 .

[5] Department for Education . Children Looked After by Local Authorities in England: Guide to the SSDA903 Collection April 2016 to 31 March 2017 . London : Department for Education , 2015 .

[6] Holmes L Soper J . Update to the Cost of Foster Care . London : Department for Education , 2010 .

[7] Viner RM Taylor B . Adult health and social outcomes of children who have been in public care: population-based study . Pediatrics 2005 ; 115**:** 894 – 99 . 15805361 10.1542/peds.2004-1311

[8] Martin A Ford T Goodman R Meltzer H Logan S . Physical illness in looked-after children: a cross-sectional study . Arch Dis Child 2014 ; 99**:** 103 – 7 . 23922058 10.1136/archdischild-2013-303993

[9] Goodman R Ford T Corbin T Meltzer H . Using the Strengths and Difficulties Questionnaire (SDQ) multi-informant algorithm to screen looked-after children for psychiatric disorders . Eur Child Adolesc Psychiatry 2004 ; 13**:** 25 – 31 . 10.1007/s00787-004-2005-3 15243783

[10] Ford T Vostanis P Meltzer H Goodman R . Psychiatric disorder among British children looked after by local authorities: comparison with children living in private households . Br J Psychiatry 2007 ; 190**:** 319 – 25 . 17401038 10.1192/bjp.bp.106.025023

[11] Meltzer H Gatward R Corbin T Goodman R Ford T . The Mental Health of Young People Looked After by Local Authorities in England . London : The Stationery Office , 2002 .

[12] Vinnerljung B Sallnäs M . Into adulthood: a follow-up study of 718 young people who were placed in out-of-home care during their teens . Child Fam Soc Work 2008 ; 13**:** 144 – 55 .

[13] Connelly G Chakrabarti M . Improving the educational experience of children and young people in public care: a Scottish perspective . Int J Incl Educ 2008 ; 12**:** 347 – 61 .

[14] Harris MS Jackson LJ O’Brien K Pecora PJ . Disproportionality in education and employment outcomes of adult foster care alumni . Child Youth Serv Rev 2009 ; 31**:** 1150 – 59 .

[15] Jackson S Martin PY . Surviving the care system: education and resilience . J Adolesc 1998 ; 21**:** 569 – 83 . 9799548 10.1006/jado.1998.0178

[16] Jones R Everson-Hock ES Papaioannou D . Factors associated with outcomes for looked-after children and young people: a correlates review of the literature . Child Care Health Dev 2011 ; 37**:** 613 – 22 . 21434967 10.1111/j.1365-2214.2011.01226.x PMC3500671

[17] Tarren-Sweeney M . The mental health of children in out-of-home care . Curr Opin Psychiatry 2008 ; 21**:** 345 – 49 . 18520738 10.1097/YCO.0b013e32830321fa

[18] Berens AE Nelson CA . The science of early adversity: is there a role for large institutions in the care of vulnerable children? Lancet 2015 ; 6736**:** 1 – 11 . 10.1016/S0140-6736(14)61131-4 PMC9594997 25638660

[19] Department for Education . Notifications of Private Fostering Arrangements: Year Ending 31 March 2014 . London : Department for Education , 2014 .

[20] Department for Education . Statistical First Release 34/2015: Children Looked After in England (Including Adoption and Care Leavers) Year Ending 31 March 2015 . London : Department for Education , 2015 .

[21] Department for Education . Outcomes for Children Looked After by Local Authorities . London : Department for Education , 2015 .

[22] Department for Education . Unique Pupil Numbers (UPN): A Guide for Schools and Local Authorities . London : Department for Education , 2013 .

[23] Department for Education . Children Looked After by Local Authorities in England: Guide to the SSDA903 Collection 1 April 2014 to 31 March 2015 . London : Department for Education , 2014 .

[24] Law J Martin EA . A Dictionary of Law . 8th edn . Oxford; UK : Oxford University Press , 2015 .

[25] Department for Education . Special Guardianship Guidance . London : Department for Education , 2005 .

[26] Department for Education . Children Looked After Return 2014 to 2015: Validation Checks . London : Department for Education , 2014 .

[27] Department for Education . Statistical First Release: Outcomes for Children Looked After by Local Authorities in England, as at 31 March 2014 . London : Department for Education , 2014 .

[28] Department for Education . Children in Need Census Matched to the National Pupil Database . London : Department for Education , 2014 .

[29] Sebba J Berridge D Luke N . The Educational Progress of Looked After Children in England: Linking Care and Educational Data . Oxford, UK : Oxford University Press , 2015 .

[30] Mc Grath-Lone L Dearden L Nasim B Harron K Gilbert R . Changes in first entry to out-of-home care from 1992 to 2012 among children in England . Child Abuse Negl 2015 ; 51**:** 163 – 71 . 26585214 10.1016/j.chiabu.2015.10.020 PMC6205623

[31] Gilbert R Fluke J O’Donnell M . Child maltreatment: variation in trends and policies in six developed countries . Lancet 2012 ; 379**:** 758 – 72 . 22169108 10.1016/S0140-6736(11)61087-8

[32] Ubbesen M-B Gilbert R Thoburn J . Cumulative incidence of entry into out-of-home care: Changes over time in Denmark and England . Child Abuse Negl 2015 ; 42**:** 63 – 71 . 25455962 10.1016/j.chiabu.2014.10.006

[33] Department for Education . Data Pack: Improving Permanence for Looked After Children . London : Department for Education , 2013 .

