# Supplementary material for: Data Resource Profile: Children Looked After Return (CLA)
Source: Int J Epidemiol. 2016 Jul 13;45(3):716–717f. doi: 10.1093/ije/dyw117 (PMC5005948; doi:10.1093/ije/dyw117)
Supplement: Supplementary Data [file dyw117_supplementary_data.zip › Supplementary_file.docx]

# Supplementary Table 1

*Placement type codes*

| **Category** | **Placement** | **Code** | **Description** | **In use**  **(year ending 31^st^ March)** |
| --- | --- | --- | --- | --- |
| Family care | Placed for adoption | A1 | Placed for adoption with parental /guardian consent (or under freeing order) not with current foster carer | 1992-2005 |
|  |  | A2 | Placed for adoption with parental /guardian consent (or under freeing order) with current foster carer | 1992-2005 |
|  |  | A3 | Placed for adoption with parental /guardian consent (or under freeing order) with current foster carer | 2006- |
|  |  | A4 | Placed for adoption with parental /guardian consent (or under freeing order) not with current foster carer | 2006- |
|  |  | A5 | Placed for adoption with placement order with current foster carer or with freeing order where parental/guardian consent was dispensed by courts | 2006- |
|  |  | A6 | Placed for adoption with placement order not with current foster carer or with freeing order where parental/guardian consent was dispensed by courts | 2006- |
|  | Foster care  (kin) | F9 | Foster placement with relative or friend | 1992-1999 |
|  |  | F1 | Foster placement with relative or friend inside Local Authority boundary | 2000-2008 |
|  |  | F4 | Foster placement with relative or friend outside Local Authority boundary | 2000-2008 |
|  |  | Q1 | Foster placement with relative or friend | 2009-2014 |
|  |  | U1 | Foster placement with relative or friend – long term fostering | 2015- |
|  |  | U2 | Foster placement with relative or friend who is also an approved adopter – FFA/concurrent planning | 2015- |
|  |  | U3 | Foster placement with relative or friend who is not long term or FFA/concurrent planning | 2015- |
|  | Foster care (stranger) | F8 | Foster placement with other foster carer | 1992-1999 |
|  |  | F2 | Foster placement with other foster carer inside Local Authority boundary, provided by Local Authority | 2000-2008 |
|  |  | F3 | Foster placement with other foster carer inside Local Authority boundary, arranged through agency* | 2000-2008 |
|  |  | F5 | Foster placement with other foster carer outside Local Authority boundary, provided by Local Authority | 2000-2008 |
|  |  | F6 | Foster placement with other foster carer outside Local Authority boundary, arranged through agency* | 2000-2008 |
|  |  | Q2 | Foster placement with other foster carer | 2009-2014 |
|  |  | U4 | Foster placement with other foster carer – long term fostering | 2015- |
|  |  | U5 | Foster placement with other foster carer who is also an approved adopter – FFA/concurrent planning | 2015- |
|  |  | U6 | Foster placement with other foster carer who is not long term or FFA/concurrent planning | 2015- |
| Group care | Children’s home | H3 | Children’s homes inside Local Authority boundary | 1992-2008 |
|  |  | H4 | Children’s homes outside Local Authority boundary | 1992-2008 |
|  |  | K2 | Children’s homes | 2009- |
|  | Residential care home | R1 | Residential care home | 1992- |
|  |  | R2 | NHS/Health Trust or other establishment providing medical or nursing care | 1992- |
|  | Residential school | S1 | All residential schools, except where dual-registered as a school and children’s home | 1992- |
|  | Other residential accommodation | H9 | Residential accommodation not subject to Children’s homes regulations but where formal support or supervision is provided | 1992-1999 |
|  |  | H5 | Residential accommodation not subject to Children’s homes regulations but where formal support or supervision is provided | 2000- |
| With parents | With parents | P1 | Placed with own parents of person with parental responsibility | 1992- |
| Other | Independent living | P2 | Independent living e.g. in flat, lodgings, bedsit, B&B or with friends, with or without formal support | 1992- |
|  |  | P3 | Residential employment including employment training and apprenticeships where accommodation is provided | 1992- |
|  | Secure unit | H1 | Secure unit inside Local Authority boundary | 1992-2004 |
|  |  | H2 | Secure unit outside Local Authority boundary | 1992-2004 |
|  |  | K1 | Secure unit | 2005- |
|  | Absent from placement† | M1 | Absent from agreed placement but whereabouts known to social services (in refuge) | 1992-2014 |
|  |  | M2 | Absent from agreed placement but whereabouts known to social services (not in refuge) | 1992-2014 |
|  |  | M3 | Whereabouts unknown | 1992-2014 |
|  | Other | R3 | Family centre or Mother and Baby unit | 1992- |
|  |  | R4 | Glenthorne Youth Treatment Centre¶ | 1992-2002 |
|  |  | R5 | Young Offender Institute or prison | 1992- |
|  |  | Z1 | Other placements | 1992- |

This table shows the current and historic placement type codes used in the Children Looked After Return and the years during which they were used. It also shows potential categories that can be created by grouping similar placements. FFA = fostering for adoption. *Care episodes recorded in CLA are funded by the state via local authorities, but may be delivered on their behalf through approved private organisations (e.g. a looked after child may be placed with an agency foster carer or in a children’s home run by a charity). †In 2014-15, additional data collection for children who were missing from care was introduced (e.g. start and end dates of missing periods) and the codes for “absent from placement” were discontinued. *^¶^*Glenthorne Youth Treatment Centre closed in 2002.

# Supplementary Table 2

*Reason looked after codes and categories of need*

| **Category of need** | **Category of need code** | **Description** (27) | **Reason looked after code** | **Description** |
| --- | --- | --- | --- | --- |
| Abuse or neglect | N1 | Children in need as a result of, or at risk of, abuse or neglect. | 10 | Preventative child welfare |
|  |  |  | 20 | Abuse or neglect |
| Child’s disability | N2 | Children and their families whose main need for services arises out of the child’s disability, illness or intrinsic condition. | 14 | Child has learning disability |
|  |  |  | 15 | Child has physical/sensory disability |
|  |  |  | 16 | Child has both physical/sensory and learning disability |
| Parental illness or disability | N3 | Children whose main need for services arises because of the capacity of their parents to care for them is impaired by disability, illness, mental illness, or addictions | 1 | Ill-health of parent(s) |
| Family in acute stress | N4 | Children whose needs arise from living in a family going through temporary crisis such that parenting capacity is diminished and some of the children’s needs are not being adequately met. | 4 | Family is homeless |
|  |  |  | 7 | Parent(s) need relief |
| Family dysfunction | N5 | Children whose needs arise mainly out of their living with families where the parenting capacity is chronically inadequate. | - |  |
| Socially unacceptable behaviour | N6 | Children and families whose need for services arise primarily out of their children’s behaviour impacting detrimentally on the community. | 21 | Risky behaviour |
|  |  |  | 22 | Child has been found guilty of an offence |
|  |  |  | 23 | Child is accused of an offence |
| Low income | N7 | Children, either living in families or independently, whose need for services arises mainly from being independent on an income below the standard state entitlements. | - |  |
| Absent parenting | N8 | Children whose need for services arises mainly from having no parents available to provide for them. Children whose parents decide it is in the best interests of the child to be adopted would be included in this category. | 2 | No parent or guardian |
|  |  |  | 3 | Abandoned or lost |
|  |  |  | 5 | Parent(s) in prison |
|  |  |  | 9 | Child aged 16+ is homeless |
|  |  |  | 11 | Adoption at request of parent(s) |
| Other* | - | Reason looked after codes that have no equivalent category of need | 8 | Child requested to be looked after |
|  |  |  | 12 | Child freed for adoption by court order |
|  |  |  | 13 | Breakdown of adoptive family |
|  |  |  | 6, 8, 19**,** 29 | Other reason (not specified) |

This table shows the reason looked after codes (used from 1^st^ April 1991 to 31^st^ March 2000) that are associated with each current category of need code. *Reasons looked after without an equivalent category of need code are categorised as “other”.

# Supplementary Table 3

*Legal status codes*

| **Category** | **Legal status** | **Code** | **Description** | **In use**  **(year ending 31^st^ March)** |
| --- | --- | --- | --- | --- |
| Care order | Interim care order | C1 | Local authority granted legal responsibility for the child for up to 28 days (must be renewed) | 1992- |
|  | Full care order | C2 | Local authority granted legal responsibility for the child (not time limited) | 1992- |
|  | Other care order | C9 | Local authority granted legal responsibility for the child | 1992-2000 |
| Placement order | Freeing order | D1 | Freeing order granted, child is freed for adoption | 1992-2005 |
|  | Placement order | E1 | Placement order granted, child is freed for adoption | 2006- |
| Child protection | Police protection powers | L1 | Child is under police protection and in Local Authority accommodation | 1992- |
|  | Emergency protection order | L2 | Child subject to emergency protection order as there are reasonable grounds for believing there is immediate risk of significant harm | 1992- |
|  | Child assessment order | L3 | Child subject to child assessment order as there are suspicions, but no firm evidence, of actual or likely significant harm | 1992- |
| Youth justice | On remand | J1 | Child is on remand, or committed for trial or sentence, and accommodated by Local Authority | 1992- |
|  | PACE detainment | J2 | Child is helping police with their enquiries and detained in Local Authority accommodation under Police and Criminal Evidence Act 1984 | 1992- |
|  | Supervision order | J3 | Child is placed under the supervision of the Local Authority and they must provide accommodation | 1992- |
| Voluntary | Single section 20 accommodation | V2 | Single period of accommodation under Section 20 of the Children Act 1989 because the child is lost or abandoned, no person has parental responsibility for them or the person caring for them cannot provide suitable accommodation or care | 1992- |
|  | Agreed series of short-term breaks* | V1 | Accommodated under an agreed series of short-term breaks | 1992-2003 |
|  |  | V3 | Accommodated under an agreed series of short-term breaks, when individual episodes of care are recorded | 2004- |
|  |  | V4 | Accommodated under an agreed series of short-term breaks, when agreements are recorded but not individual episodes of care | 2004- |

This table shows current and historic legal status codes used in the Children Looked After Return and the years during which they were used. It also shows potential categories that can be created by grouping similar legal statuses. *Short term break codes are used for children who are looked after for respite reasons.

# Supplementary Table 4

*Reason episode ceased codes*

| **Category** | **Reason episode ceased** | **Code** | **Description** | **In use**  **(year ending 31^st^ March)** |
| --- | --- | --- | --- | --- |
| Child returned home | Return home | E4 | Return home to live with parents, relatives, or other person with parental responsibility | 1992-2015 |
|  | Planned return home | E4A | Return home to live with parents, relatives, or other person with parental responsibility as part of the care planning process | 2015- |
|  | Unplanned return home | E4B | Return home to live with parents, relatives, or other person with parental responsibility which was not as part of the care planning process | 2015- |
| Other exits to a family setting | Adopted | E1 | Adopted | 1992-2006 |
|  |  | E11 | Adopted – application for an adoption order unopposed | 2007- |
|  |  | E12 | Adopted – consent dispensed by court | 2007- |
|  | Special guardianship order | E42 | Special guardianship made | 2005-2006 |
|  |  | E43 | Special guardianship made to former foster carers | 2007- |
|  |  | E44 | Special guardianship made to carers other than former foster carers | 2007- |
|  | Residence order | E41 | Residence order (or, from 22 April 2014, a child arrangements order which sets out with whom the child is to live) granted. | 2005- |
| Independent exits | Independent living  (aged 16-18) | E5 | Moved into independent living arrangement and no longer looked after: supportive accommodation providing formalised advice/support arrangements (e.g. most hostels, YMCAs, foyers, and care leavers projects) | 1992- |
|  |  | E6 | Moved into independent living arrangement and no longer looked after : accommodation providing no formalised advice/support arrangements (e.g. bedsit, own flat, living with friends) | 1992- |
|  | Exit as an adult | E13 | Left care to live with parents, relative, friends or other person with no parental responsibility (i.e. on/after 18^th^ birthday) | 2015- |
|  |  | E15 | Age assessment determined child is aged 18 or over and E5, E6 and E7 do not apply (e.g. an unaccompanied asylum seeking child whose age has been disputed) | 2015- |
| Care transfers | Children’s social care | E3 | Care taken over by other local authority in the UK | 1992- |
|  | Adult social care | E7 | Transferred to residential care funded by Adult Social Services | 1992- |
| Youth justice related exits | Entered custody | E9 | Sentenced to custody, ceased to be looked after. | 1992- |
|  | Left custody | E14 | Accommodation on remand ended | 2015- |
| Other exits | Died | E2 | Died | 1992- |
|  | Moved abroad | E16 | Child moved abroad | 2015- |
|  | Other | E8 | Period of being looked after ceased for any other reason | 1998- |

This table shows current and historic reason episode ceased codes used in the Children Looked After Return and the years during which they were used. It also shows potential categories that can be created by grouping similar exits.
